# LGBTQ+ healthcare preparedness among undergraduate medical students in India: a cross-sectional study of awareness, attitudes, and clinical confidence

**DOI:** 10.1186/s12909-026-10138-5

**Published:** 2026-08-13

**Authors:** Pratham Mehta, Vihitha Vunnam, Smitha Rani, Sharanya Bose, Sona Sudhi, Bindu A

**Affiliations:** 1 Department of Anatomy, JSS Medical College, JSSAHER, Mysuru, India; 2 JSS Medical College, JSSAHER, Mysuru, India; 3 Department of Forensic Medicine and Toxicology, JSS Medical College, JSSAHER, Mysuru, India; 4 Department of Psychiatry, JSS Medical College, JSSAHER, Mysuru, India

**Keywords:** LGBTQ+ healthcare, Medical education, Competency-based medical education, Awareness, Attitude, Confidence, Mediation analysis, India

## Abstract

**Background:**

Lesbian, Gay, Bisexual, Transgender, Queer (LGBTQ+) individuals in India experience significant healthcare disparities, including higher burdens of mental health conditions, HIV, and stigma, and frequently encounter discrimination in healthcare settings. Yet the Indian National Medical Commission’s Competency-Based Medical Education (CBME) curriculum contains no explicit LGBTQ+ health competencies. Prior studies have typically measured awareness, attitudes, and confidence independently, but whether attitudes link awareness to clinical confidence remains unexplored, particularly in CBME-trained cohorts in low- and middle-income countries. This study aimed to assess awareness, attitudes, and self-reported confidence in LGBTQ+ healthcare among undergraduate medical students, examine relationships between these domains across academic years, and test whether attitudes mediate the awareness–confidence pathway.

**Methods:**

An analytical cross-sectional study was conducted among 229 undergraduate MBBS (Bachelor of Medicine, Bachelor of Surgery; the primary undergraduate medical degree in India) students with at least six months of clinical exposure at a private medical college in South Karnataka, India (November 2024–January 2025). Data were collected using a structured questionnaire assessing awareness (31 items), attitudes (10 items), and confidence (6 items), adapted from a previously published instrument (Cronbach’s α: awareness = 0.95, attitude = 0.80, confidence = 0.81). Domain scores were summarised as median and interquartile range (IQR). Differences across academic years were examined using the Kruskal–Wallis test followed by Dunn’s post hoc test with Holm adjustment. Associations between domains were assessed using Spearman’s rank correlation. Multivariable linear regression was performed to identify independent predictors of confidence. Exploratory mediation analysis examined whether attitudes mediated the relationship between awareness and confidence.

**Results:**

Students demonstrated moderate awareness (3.59 [3.03–4.06]) and generally favourable attitudes (3.40 [3.00–3.80]), but comparatively lower confidence (3.17 [2.83–3.67]). Awareness increased significantly with advancing academic year (χ²(2) = 38.32, *p* < 0.001), whereas attitudes (*p* = 0.522) and confidence (*p* = 0.919) did not. In multivariable analysis, awareness (B = 0.446, 95% CI: 0.320–0.572), attitudes (B = 0.414, 95% CI: 0.289–0.539), and prior training (B = 0.245, 95% CI: 0.169–0.320) were independently associated with higher confidence (all *p* < 0.001). Notably, after adjustment, third-year (B = − 0.256, *p* = 0.005) and final-year students (B = − 0.275, *p* = 0.009) demonstrated significantly lower confidence than second-year students. Exploratory mediation analysis demonstrated that attitudes partially mediated the awareness–confidence relationship (indirect effect = 0.145, 95% CI: 0.079–0.222; proportion mediated: 27.1%, *p* < 0.001).

**Conclusions:**

To our knowledge, this is among the first studies to examine LGBTQ+ healthcare preparedness in a CBME-trained medical student cohort in India. Although awareness improved with academic progression, this did not translate into greater clinical confidence. The lower confidence among senior students after adjustment for awareness may reflect a curricular gap within CBME, whereby increasing clinical exposure occurs without the structured mechanisms needed to build applied competence. Attitudes accounted for only about a quarter of the awareness–confidence association, indicating that attitudinal change alone is unlikely to be sufficient without direct skills-based training. These findings support the integration of structured, assessed LGBTQ+ health content, including within the AETCOM framework, into the CBME curriculum.

**Supplementary Information:**

The online version contains supplementary material available at https://doi.org/10.1186/s12909-026-10138-5.

## Background

Sexual and gender minority populations in India face a disproportionate burden of mental health disorders, HIV, stigma, and structural barriers to accessing care, yet evidence on how to address these disparities through medical education remains limited [1]. Despite the decriminalisation of same-gender sexual relationships in India under Sect. 377 of the Indian Penal Code in 2018, subsequent legal developments including the Supreme Court’s 2023 decision declining to legalise same-sex marriage indicate that structural and social challenges persist, and Lesbian, Gay, Bisexual, Transgender, Queer (LGBTQ+) individuals continue to face barriers in receiving capable, compassionate, and non-judgmental healthcare [2–4]. Fear of discrimination and prior negative healthcare experiences lead many LGBTQ+ individuals to delay or avoid seeking medical care [5]. The LGBTQ+ population experiences higher rates of mental health disorders, substance abuse, and unmet healthcare needs, largely due to stigma and gaps in provider training [6].

LGBTQ+ health has historically been overlooked in medical curricula across both developed and developing nations. Undergraduate medical education has been shown to provide limited structured training in LGBTQ+ health, contributing to inadequate preparedness among future healthcare providers [7]. More recent studies continue to demonstrate gaps in knowledge, attitudes, and confidence, suggesting that this limitation persists [8]. This gap is likely more pronounced in India, where no explicit curricular mandate exists. India’s National Medical Commission (NMC) and its Competency-Based Medical Education (CBME) curriculum do not explicitly include LGBTQ+ health as a distinct area of instruction [9]. Furthermore, some medical textbooks have been criticised for pathologising queer identities and lacking an evidence-based approach to LGBTQ+ health [10]. 

Preparedness for LGBTQ+ healthcare is commonly characterised across three related domains: awareness, reflecting recognition-level knowledge; attitudes, reflecting the values and orientation that shape engagement with care; and confidence, reflecting a learner’s self-efficacy, or perceived readiness to perform clinical tasks. Indian studies have examined these domains largely in isolation, generally reporting favourable or accepting attitudes among medical students alongside inconsistent knowledge and limited structured training, particularly in transgender health [11–13]. Elsewhere, these domains have been assessed together like in the UK medical-student survey from which our instrument is adapted, which evaluated both knowledge of and self-rated competence in LGBTQ+ health [14], but such work has rarely been conducted in CBME-based systems in low- and middle-income countries, and has seldom examined how the domains relate to one another. It therefore remains unclear whether rising awareness during training is accompanied by more favourable attitudes and greater clinical confidence, and through what pathway knowledge translates into clinical preparedness.

In the absence of formal instruction, students may rely on informal sources such as stereotypes, media representations or peer discussions, which can perpetuate misinformation. In the Indian context, where sociocultural conservatism and limited institutional support for LGBTQ+ inclusion persist, there is a need to evaluate how current medical training prepares students to address the healthcare needs of LGBTQ+ patients. To address this gap, the present study aimed to assess undergraduate medical students’ awareness, attitudes, and self-reported confidence in providing care to LGBTQ+ individuals, and to examine how these domains relate to and influence one another.

## Methods

### Study design and setting

This analytical cross-sectional study was conducted at a medical college in South Karnataka, India. after Institutional Ethics Committee approval (Approval No: JSSMC/IEC/070324/15 NCT/2024-25; Date: 02 May 2024). Reporting followed the Strengthening for Reporting of Observational Studies in Epidemiology (STROBE) guidelines for cross-sectional studies [15]. The completed STROBE checklist is provided in Additional file 1. Data were collected between November 2024 and January 2025 during breaks between scheduled academic sessions.

### Participants

All undergraduate MBBS (Bachelor of Medicine, Bachelor of Surgery; the primary undergraduate medical degree in India) students with at least six months of clinical exposure (second-, third-, and final-year students) who were trained under the CBME curriculum were eligible. These correspond to different phases of the CBME curriculum. This threshold was chosen to ensure that participants had sufficient clinical exposure to patient interactions and clinical environments, which are necessary for meaningful assessment of confidence in LGBTQ+ healthcare-related tasks. First-year students (Phase I) were excluded due to insufficient clinical exposure. The link to the questionnaire was distributed via the respective class groups to maximise participation. Participation was voluntary and anonymous, and no incentives were offered. Only complete responses were analysed; the online form required all items to be answered, so the analysed dataset contained no missing data and no imputation was required. A single-centre convenience sampling approach was used to ensure feasibility and maximise participation within the available study period.

A sample size was calculated using finite population correction, assuming a 95% confidence level, 5% margin of error, and maximum variability (*p* = 0.5). Based on an eligible population of 465 students, the minimum required sample size was 211. The questionnaire was circulated to all 465 students present in class during the data collection period, and repeated reminders were sent through official student communication channels to improve participation and minimise non-response. A total of 229 complete responses were obtained, exceeding the minimum required sample size and corresponding to a response rate of 49.2%.

### Questionnaire and measures

The questionnaire was adapted from excerpts of a previously used questionnaire (with permission) reported by Parameshwaran et al. [14] In this study we distinguish related constructs. Awareness refers to recognition-level familiarity with LGBTQ+ terminology and health concepts; attitudes refer to evaluative orientations toward LGBTQ+ individuals and their care; and confidence refers to self-efficacy which is a learner’s perceived capability to perform a clinical task, rather than demonstrated competence in practice [16]. Preparedness is used as an umbrella term spanning these domains. The adapted questionnaire comprised three domains: Attitude (10 items; five-point Likert scale ranging from 1 = strongly disagree to 5 = strongly agree), Confidence (6 items; ranging from 1 = not at all confident to 5 = very confident, assessing clinical tasks such as sexual-history taking and ward allocation), and Awareness (31 items rated from 1 = never heard to 5 = very familiar, assessing commonly used LGBTQ+ terms). For each domain, item responses were aggregated at the participant level by calculating the mean of all items within that domain, resulting in continuous domain scores ranging from 1 to 5, with higher scores indicating greater awareness, more favourable attitudes, and higher confidence. Negatively worded items were reverse coded prior to aggregation so that higher scores consistently indicated more favourable attitudes. The complete adapted questionnaire used in this study is provided as Additional file 2.

The original instrument [14] assessed UK medical students’ knowledge of and self-rated competence in LGBTQ+ health across domains including mental and sexual/reproductive health. The attitude and confidence domains were retained with minor modifications, whereas the awareness domain was reframed: the original items rated confidence in understanding LGBTQ+ terms, whereas the adapted items assess self-reported familiarity (for example, rather than rating confidence in explaining a term such as ‘cisgender’, participants rated how familiar they were with it). This better matched our objective of measuring recognition-level knowledge, though it limits direct comparability with Parameswaran et al. Items on clinical practices, observed behaviours, and participants’ sexuality were excluded as these fell outside the study objectives. Prior training was assessed with a single self-reported item asking whether participants had received prior exposure to LGBTQ+ health-related teaching, rated on a five-point Likert scale from strongly disagree to strongly agree.

Because the source instrument was developed in a UK context, the adapted questionnaire was reviewed by the study team for content and contextual relevance to the Indian setting. It was then pilot-tested (*n* = 26) to confirm clarity, face validity, and internal consistency, which was acceptable across all three domains (Cronbach’s α: awareness = 0.95, attitude = 0.81, confidence = 0.75); pilot data were excluded from the final analysis. Given the exploratory aim and the pilot sample size, validation focused on content and face validity and internal consistency rather than latent-structure methods such as factor analysis; internal consistency was reassessed in the final dataset (reported in the Results). Responses were collected using Google Forms and downloaded directly into a secure database for analysis.

### Statistical analysis

Descriptive statistics were used to summarise participant characteristics and study variables. Continuous domain scores were reported as median (interquartile range [IQR]) and categorical variables as frequencies and percentages. Group comparisons by year of study were performed using the Kruskal–Wallis test. Where statistically significant differences were observed, post hoc pairwise comparisons were conducted using Dunn’s test with Holm adjustment for multiple comparisons. Associations between domain scores were assessed using Spearman’s rank correlation coefficient. Multivariable linear regression analysis was performed to identify independent predictors of confidence, adjusting for gender, year of study, and prior training. Domain scores were computed as the mean of multiple Likert-type items, yielding composite scores that approximate an interval scale and are commonly analysed parametrically, whereas single ordinal items are better suited to non-parametric tests. We therefore used non-parametric tests for unadjusted group comparisons and linear regression for adjusted modelling of the composite confidence score [17]. Linear regression was preferred over ordinal alternatives such as ordinal logistic regression because the composite confidence score is a near-continuous mean rather than a single ordered category; modelling it ordinally would require collapsing it into arbitrary bands and discard information, whereas linear regression preserves the full score and yields directly interpretable coefficients. Although Shapiro–Wilk testing indicated deviations from normality, linear regression is robust to moderate non-normality at this sample size, and residual diagnostics (Q–Q plots) supported its use [18]. An exploratory mediation analysis was conducted using the mediation package in R, with 5000 quasi-Bayesian Monte Carlo simulations to estimate the indirect, direct, and total effects. Awareness was specified as the antecedent, attitudes as the mediator, and confidence as the outcome on conceptual grounds, reflecting a hypothesised sequence in which foundational knowledge may shape evaluative attitudes, which in turn may support task-specific confidence. Both models were adjusted for potential confounders, including gender, year of study, and prior training. Given the cross-sectional design, mediation analysis was conducted as an exploratory exercise and should not be interpreted as evidence of causal mediation. All analyses were two-tailed, with statistical significance set at *p* < 0.05. Analysis was performed using R (version 4.5.2).

## Results

### Participant characteristics

Of the eligible participants, 229 participants responded and were included in the final analysis. The majority of participants were female (62.4%), followed by males (36.7%), and a small proportion (0.9%) preferred not to disclose gender. Participants were distributed across years of study as follows: second year (43.7%), third year (32.3%), and final year (24.0%). A majority of participants (68.6%) reported no prior exposure to LGBTQ+ health content, 21.4% were neutral, and only 10.0% (Table 1).

**Table 1:** Participant characteristics (*N* = 229)

Variable	Category	*n* (%)
Gender	Female	143 (62.4)
	Male	84 (36.7)
	Prefer not to say	2 (0.9)
Year of Study	Second year	100 (43.7)
	Third year	74 (32.3)
	Final year	55 (24.0)
Perceived Adequacy of Prior Training*	Inadequate	157 (68.6)
	Neutral	49 (21.4)
	Adequate	23 (10.0)

* Prior-training responses were collected on a 5-point Likert scale (strongly disagree to strongly agree) and collapsed as: strongly disagree/disagree = Inadequate; neutral = Neutral; agree/strongly agree = Adequate

### Domain scores and item-level findings

Median domain scores were moderate across all domains. Awareness had a median score of 3.59 (IQR 3.03–4.06), attitude 3.40 (IQR 3.00–3.80), and confidence 3.17 (IQR 2.83–3.67) (Table 2), indicating relatively favourable attitudes but comparatively lower confidence. Internal consistency of the questionnaire domains was assessed using Cronbach’s alpha. The awareness scale demonstrated excellent reliability (α = 0.95), while the attitude (α = 0.80) and confidence (α = 0.81) scales showed good internal consistency (see Additional file 3: Table A1).

**Table 2:** Descriptive statistics of key study domains (Median [IQR])

Domain	Median (IQR)	Range
Awareness	3.59 (3.03–4.06)	1.00–5.00
Attitude	3.40 (3.00–3.80)	1.40–5.00
Confidence	3.17 (2.83–3.67)	1.00–5.00

Item-level analysis revealed generally favourable attitudes, with 85.6% of participants agreeing that LGBTQ+ patients deserve equal quality care and 70.3% expressing comfort being known as providers for LGBTQ+ patients. However, responses were mixed on items concerning patient disclosure, with 58.9% disagreeing that patients should always disclose their sexual orientation. Awareness varied across concepts, with high familiarity for common terms such as ‘gay’ and ‘lesbian’ (approximately 80%), but lower familiarity for less commonly encountered terms such as ‘polyamorous’ (42.3%). Confidence was comparatively lower for clinical decision-making tasks, with only 24.5% reporting confidence in determining ward placement for transgender patients and 21.4% in identifying LGBTQ+-specific health services, although communication-related tasks showed relatively higher confidence. Detailed item-wise response distributions, including the percentage of responses for each response category for all questionnaire items, are presented in Additional file 3: Table A2.

### Comparison across academic years

**Fig. 1:**
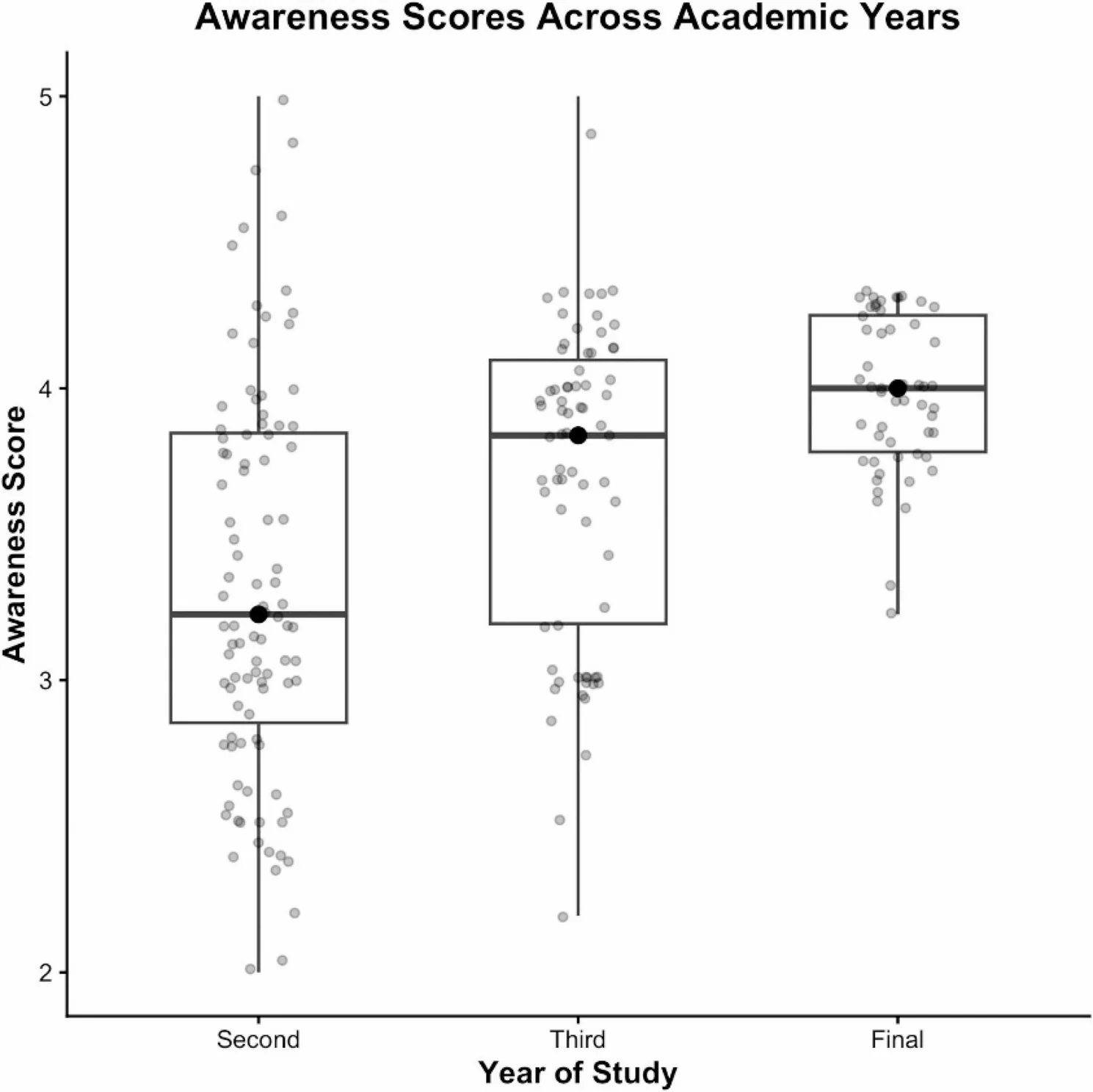
Boxplot depicting distribution of awareness scores across academic years

Awareness scores differed significantly across academic years, increasing progressively from second to final year (*p* < 0.001), whereas attitude (*p* = 0.522) and confidence (*p* = 0.919) did not (Table 3). Post hoc comparisons confirmed significantly higher awareness at each successive year (all adjusted *p* ≤ 0.003; Additional file 3, Table A3), as illustrated in Fig. 1.

**Table 3:** Non-parametric comparison of domain scores across year of study (Median [IQR])

Outcome	Second Year	Third Year	Final Year	χ² (df = 2)	*p*-value
Awareness	3.23 (2.85–3.85)	3.84 (3.03–4.06)	4.00 (3.78–4.25)	38.32	< 0.001
Attitude	3.30 (3.08–3.70)	3.50 (3.00–3.80)	3.40 (3.00–3.98)	1.30	0.522
Confidence	3.17 (2.83–3.67)	3.17 (3.00–3.50)	3.17 (2.83–3.67)	0.17	0.919

### Correlations, model assumptions and multivariable analysis

**Fig. 2:**
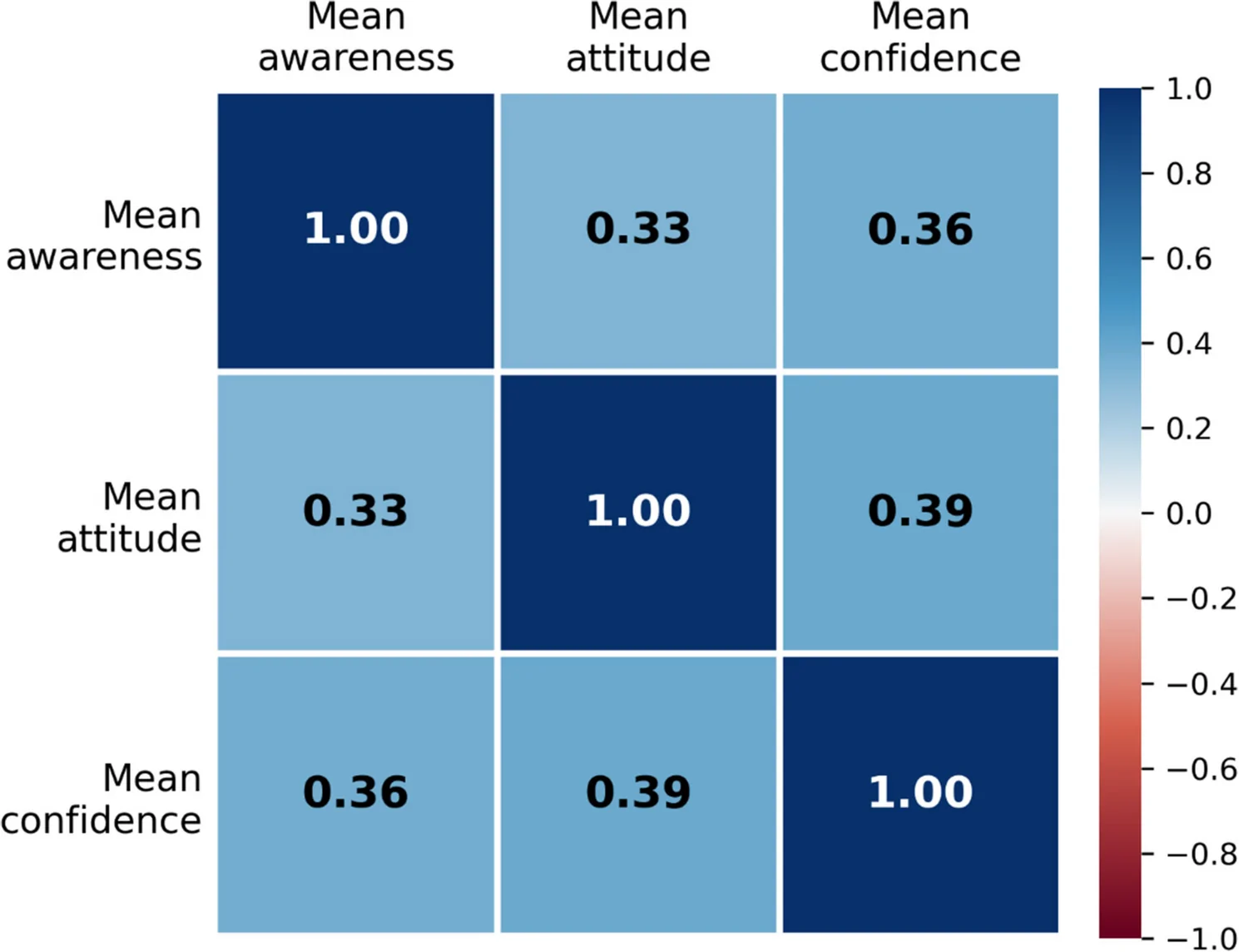
Spearman rank correlation matrix among study domains

Spearman rank correlation analysis demonstrated statistically significant positive monotonic associations among all domains. Awareness was moderately correlated with confidence (*ρ* = 0.44, *p* < 0.001) and weakly correlated with attitude (*ρ* = 0.28, *p* < 0.001). Attitude was also moderately correlated with confidence (*ρ* = 0.42, *p* < 0.001) (Fig. 2).

Prior to multivariable analysis, model assumptions were evaluated. Shapiro–Wilk testing indicated statistically significant deviations from normality across all domain scores; however, visual inspection of Q–Q plots demonstrated that deviations were primarily confined to the distribution tails, with central portions approximating normality (see Additional file 3: Figure A1 and Tables A4–A5). An integrated assessment combining statistical tests, visual diagnostics, and residual analysis supported the suitability of linear regression for adjusted analyses. Multicollinearity was not observed, with GVIF¹ᐟ²ᴰᶠ values ranging from 1.03 to 1.14, indicating negligible collinearity (see Additional file 3: Table A6).

In univariable analysis, awareness, attitude, and prior training were each significantly associated with higher confidence, whereas gender and year of study were not (Table 4).

**Table 4:** Univariable and Multivariable Linear Regression Analysis for predicting confidence

Predictor	Univariable B (95% CI)	*p*-value	Multivariable B (95% CI)	*p*-value
Awareness score	0.488 (0.359–0.616)	< 0.001	0.446 (0.320–0.572)	< 0.001
Attitude score	0.576 (0.434–0.719)	< 0.001	0.414 (0.289–0.539)	< 0.001
Prior training score	0.205 (0.114–0.296)	< 0.001	0.245 (0.169–0.320)	< 0.001
Male vs. Female	0.030 (− 0.169–0.229)	0.767	0.082 (− 0.080–0.243)	0.320
Other vs. Female	0.371 (− 0.661–1.403)	0.479	0.300 (− 0.498–1.098)	0.459
Third year vs. Second year	−0.016 (− 0.239–0.206)	0.887	−0.256 (− 0.435–−0.077)	0.005
Final year vs. Second year	0.034 (− 0.209–0.278)	0.782	−0.275 (− 0.480–−0.070)	0.009

Model fit: adjusted R^2^= 0.406; F(7, 221) = 23.28, *p* < 0.001

In the multivariable model, awareness, attitude, and prior training remained independently associated with higher confidence, while gender was not (Table 4). Notably, third- and final-year students showed significantly lower confidence than second-year students after adjustment (both *p* ≤ 0.009), despite no crude difference in confidence across years (Table 3). This divergence between the unadjusted and adjusted results is consistent with negative confounding (a suppression effect): because awareness rose steeply with academic year and was itself strongly and positively associated with confidence, the crude comparison masked an underlying negative association between seniority and confidence. Once awareness and attitude were held constant, this association was unmasked, indicating that at a comparable level of awareness, senior students reported lower confidence than juniors.

### Exploratory mediation analysis

Exploratory mediation analysis demonstrated a significant indirect effect of awareness on confidence through attitude (ACME = 0.145, 95% CI: 0.079–0.222, *p* < 0.001), indicating partial mediation (Table 5). The direct effect of awareness on confidence remained significant after accounting for attitude (ADE = 0.387, 95% CI: 0.261–0.513, *p* < 0.001). The total effect of awareness on confidence was 0.532 (95% CI: 0.400–0.665, *p* < 0.001), with approximately 27.1% of the effect mediated through attitude.

**Table 5:** Exploratory mediation analysis: awareness → attitude → confidence

Effect	Estimate	95% CI	*p*-value
ACME (indirect effect)	0.145	0.079–0.222	< 0.001
ADE (direct effect)	0.387	0.261–0.513	< 0.001
Total effect	0.532	0.400–0.665	< 0.001
Proportion mediated	27.1%	15.8% to 41.4%	< 0.001

*ACME* Average Causal Mediation Effect, *ADE* Average direct effect

## Discussion

In this cohort, attitudes toward LGBTQ+ individuals were generally favourable and awareness moderate, whereas self-reported clinical confidence was comparatively lower. These findings are consistent with other Indian studies. Waghmare et al. (2024) reported that roughly two-thirds of students expressed supportive attitudes, alongside only partially satisfactory knowledge (≈ 61%) and an absence of structured clinical training [11]. Notably, some Indian studies report more satisfactory self-rated competence among healthcare trainees [13], underscoring variability across settings and training levels. Divya and Menon (2022) described a curricular vacuum in transgender health, where even final-year students received little structured training [12]. The present study adds to this evidence by demonstrating a similar pattern within a CBME-trained cohort, suggesting that existing curricular reforms have not yet adequately addressed this gap.

Awareness of LGBTQ+ health was higher among students in later academic years, whereas clinical confidence was not, consistent with a gap between conceptual knowledge and clinical preparedness. While attitudes may be shaped through sociocultural exposure, clinical confidence requires structured opportunities for skills acquisition and supervised practice. In the absence of such an environment, knowledge and attitudes may not equate to clinical confidence, a pattern also observed in previous studies [14]. 

The observed positive correlations between awareness, attitudes, and confidence indicate that these domains are interconnected components of preparedness. While overall attitude scores were favourable, item-level responses revealed uncertainty on specific issues, such as patient disclosure of sexual orientation, suggesting that favourable mean scores may mask important variability in understanding. The progressive rise in awareness across academic years may partly reflect the ‘hidden curriculum’, whereby students acquire familiarity with LGBTQ+ terminology informally; our data cannot confirm this mechanism but show that awareness rose without corresponding gains in confidence [19]. Importantly, this increase appears to occur at the level of recognition rather than applied understanding: the awareness domain assessed self-reported familiarity with terminology rather than applied clinical reasoning, so rising scores reflect greater recognition of terms rather than a demonstrated ability to use them in clinical decision-making.

In contrast, confidence did not improve with advancing academic year, indicating that seniority alone does not ensure preparedness for LGBTQ+ healthcare. Notably, after adjustment for awareness, attitudes, and prior training, third- and final-year students demonstrated significantly lower confidence scores than second-year students. This negative association emerged only after adjustment, suggesting that rising awareness may have masked a persisting gap in applied confidence among senior students. This may reflect a curricular gap within CBME, where progressive clinical exposure increases awareness of LGBTQ+ terminology without providing the structured mechanisms to develop corresponding clinical skills. The absence of explicit LGBTQ+ competencies within the CBME guidelines may mean that students encounter increasing clinical complexity without the background required to build confidence. However, given the cross-sectional design, this interpretation should be considered with caution.

This pattern is open to more than one explanation. The most likely, in our view, is a Dunning–Kruger-type effect, in which junior students overestimate their capability before clinical exposure reveals the complexity of LGBTQ+ care; later scores may therefore reflect improved self-calibration rather than a true decline in ability [20]. This is compounded by limited opportunity for the experiential learning through which clinical confidence is built. Experiential and situated learning theories [21, 22], together with Bandura’s social cognitive theory [16], hold that self-efficacy develops through mastery experiences, observation, and structured feedback rather than knowledge acquisition alone. In LGBTQ+ healthcare, such experiences might include supervised history-taking with LGBTQ+ patients, observing clinicians’ model inclusive care, and feedback that reinforces appropriate clinical behaviour. Consistent with this, educational approaches using real-case discussion, role-play, and direct encounters with LGBTQ+ individuals have improved self-confidence, comfort, and clinical preparedness across settings [23]. From this perspective, rising awareness without a corresponding rise in confidence is consistent with a curriculum that builds familiarity with terminology while offering few opportunities for supervised practice.

Prior training emerged as an independent predictor of confidence, suggesting that structured educational exposure plays a meaningful role in improving preparedness over and above incidental clinical exposure. While students may encounter LGBTQ+ concepts informally through clinical environments or peer discussions, such learning is often unstructured and insufficient for skill development. Within CBME frameworks, clinical competence is achieved through guided practice and assessment; however, in the absence of LGBTQ+-specific content, these mechanisms are not effectively applied to this domain. The consistent association between prior training and confidence across all analyses may suggest that even brief, structured interventions could produce meaningful gains — a finding with direct implications for curriculum design. This aligns with international evidence: a multi-centre study of about 1700 healthcare professional students across the United States found both patient contact and formal curricular hours to independently predict LGBTQ+ cultural competence [24]. The relatively lower confidence after adjustment observed in the present study may reflect lack of structured exposure within Indian undergraduate curricula.

Because the design is cross-sectional and awareness, attitudes, and confidence were measured concurrently, these mediation estimates are exploratory and hypothesis-generating: they decompose associations rather than establish temporal order or causal direction, and self-efficacy may equally shape attitudes. With this caveat, awareness and attitudes appeared interrelated in shaping confidence: attitudes accounted for a meaningful share of the awareness–confidence association, approximately 27.1% — consistent with a potential mediating role in which greater awareness is linked to more favourable attitudes, which in turn support higher confidence. The larger remaining share was direct, indicating that attitudes did not fully account for the observed association. Longitudinal designs tracking the same cohort across training would be needed to confirm this pathway and clarify whether attitudes genuinely mediate the awareness–confidence relationship.

In low- and middle-income countries such as India, these gaps carry broader implications. LGBTQ+ individuals frequently experience discrimination, misgendering, and barriers to accessing care, all of which erode trust in healthcare systems [25]. A multinational survey found that 69% of medical students had no specific LGBTQ+ training, yet 85% expressed a desire for such education [8]. Without adequate preparation, graduates may unintentionally perpetuate these disparities, which include mental health challenges, heightened HIV risk, delayed care-seeking, and limited access to inclusive services [26]. 

### Implications for curriculum reform

The findings of this study suggest that although students demonstrate favourable attitudes toward LGBTQ+ healthcare, they are not adequately prepared to translate these attitudes into practice. This gap is most evident in the domain of confidence, despite relatively high levels of awareness. Integrating structured LGBTQ+ health content into the CBME curriculum may help bridge this gap. Given that attitudes were generally favourable while confidence remained comparatively low, the priority may not be attitudinal change itself, but rather the development of explicit, assessable competencies focused on the application of knowledge and skills, such as conducting an inclusive sexual history, using affirming terminology, ensuring appropriate ward allocation for transgender patients, and facilitating referral to LGBTQ+-relevant services. Within a competency-based framework, such capabilities are best developed not through additional content alone but through defined learning outcomes, supervised workplace-based practice, and assessment that requires learners to demonstrate them. Although situated within India’s CBME framework, this attitude–confidence gap is unlikely to be unique to this setting: wherever curricula build awareness of minority health without embedding assessed, skills-based practice, favourable attitudes alone are unlikely to translate into clinical confidence. The principles below, though framed for the Indian context, may therefore inform LGBTQ+ curricular reform more broadly.

The key implications for curriculum reform are summarised in Table 6. They follow from three findings of this study — rising awareness without corresponding gains in confidence, favourable attitudes alongside lower applied confidence, and prior training as the strongest modifiable predictor of confidence — supplemented, where indicated, by the broader medical-education literature and the authors’ educational experience.

**Table 6:** Implications for curriculum reform

• Introduce LGBTQ+ health early, within foundational professionalism and communication teaching (in India, the AETCOM* module), framed as equitable, evidence-based care rather than a matter of ethical debate. Integrating terminology, health disparities, and legal context before clinical exposure addresses the low baseline preparedness and reliance on informal learning observed in this cohort.
• Prioritise skills-based, experiential learning : case discussions, role-play, and simulated encounters such as inclusive history-taking and gender-affirming care. Because awareness rose across academic years while confidence did not, recognition-level knowledge alone appears insufficient to build applied confidence.
• Incorporate LGBTQ+-inclusive assessment, including OSCE stations on inclusive history-taking, correct terminology, ward allocation for transgender patients, and referral; the domains in which students reported the lowest confidence.
• Invest in faculty development, enabling educators to model affirming care and provide feedback during routine teaching. That confidence did not rise with seniority, and was lower among senior students after adjustment, suggests the modelling and feedback that build self-efficacy are currently lacking; this recommendation is also supported by the broader medical-education literature.
• Emphasise applied capability over attitudinal content, as attitudes were already favourable while confidence lagged, and attitudes accounted for only part of the awareness–confidence association.
Even brief, structured educational interventions can produce measurable improvements in knowledge, attitudes, and confidence. The strong association between prior training and confidence in this study suggests that the primary barrier to preparedness is absence of structured exposure rather than student unwillingness, a barrier likely shared by any system where such training is not yet embedded.

*AETCOM Attitude, Ethics and Communication- a longitudinal competency-based module in India's undergraduate medical curriculum

### Strengths and limitations

This study has several strengths, including being among the first studies assessing LGBTQ+ healthcare preparedness in a CBME-trained group and the use of a questionnaire adapted from a previously published instrument with demonstrated use in similar populations and inclusion of students across different stages of training, allowing meaningful comparison between academic years. The sample size was adequate for an exploratory cross-sectional study. In addition, the use of multivariable regression and exploratory mediation analysis enabled exploratory examination of the relationships between awareness, attitude, and confidence, although these analyses remain associational given the cross-sectional design.

However, certain limitations should be considered. The instrument was adapted from a UK questionnaire reportedly informed by local LGBTQ+ community input, so the extent to which all items capture preparedness in the Indian context remains uncertain; relatedly, a few attitude items on disclosure and access to care may reflect heterogeneous reasoning about patient safety, autonomy, or access rather than a single underlying attitude, and their item-level findings should be read with this in mind. As the awareness domain was reframed from the source instrument, its latent structure was not formally re-validated in this population; the reliability observed supports internal consistency but not structural equivalence, which future work could establish. Because participation was voluntary and based on convenience sampling, students more interested in or comfortable with LGBTQ+ health may have been likelier to respond, introducing self-selection bias that could inflate awareness and attitude estimates. Self-reported data may also be subject to social desirability bias, which could have inflated favourable attitude scores. Self-reported confidence may further reflect factors beyond preparedness — willingness to engage in LGBTQ+ care, personal values, or implicit bias — so low confidence should not be read solely as a training deficit. As a single-centre study, the findings may not be generalisable, although the nationally mandated CBME framework suggests structural similarities across Indian medical colleges; and the cross-sectional design cannot establish causal relationships, so the mediation analysis should be considered exploratory.

Future work should incorporate learner and other stakeholder perspectives like qualitative exploration of which educational experiences students perceive as most effective for building confidence in LGBTQ+ care, which could validate these findings and help explain why favourable attitudes alone did not account for confidence.

## Conclusion

In summary, while undergraduate medical students demonstrated favourable attitude and moderate awareness of LGBTQ+ healthcare, confidence in applying this knowledge was limited. These findings suggest that exposure alone may not be sufficient; structured, skills-based teaching is required to develop clinical preparedness. That attitudes accounted for only part of the awareness–confidence association suggests that attitudinal improvement, while important, is unlikely to be sufficient on its own, and that direct skills-based training is also needed to bridge the gap between knowledge and clinical competence. Addressing these gaps is important for reducing healthcare disparities and improving patient outcomes among LGBTQ+ populations. Incorporating structured LGBTQ+ content into the CBME curriculum, anchored within existing frameworks such as AETCOM, may better equip future doctors to provide respectful, competent, and equitable care to sexual and gender minority patients.

## Supplementary Information

Additional file 1.

Additional file 2.

Additional file 3.

## Data Availability

The datasets generated and/or analyzed during the current study are available from the corresponding author on reasonable request as per institutional sharing guidelines.

## References

[1] Chakrapani V, Newman PA, Shunmugam M, Rawat S, Mohan BR, Baruah D, et al. A scoping review of lesbian, gay, bisexual, transgender, queer, and intersex (LGBTQI+) people’s health in India. PLOS Glob Public Health. 2023;3(4):e0001362. 10.1371/journal.pgph.0001362. PMC10118178 37079524

[2] Rao TSS, Jacob KS. Homosexuality and India. Indian J Psychiatry. 2012;54(1):1. 10.4103/0019-5545.94636. PMC3339212 22556428

[3] Navtej Singh Johar v. Union of India. Writ Petition (Criminal) No. 76 of 2016. Supreme Court of India. 2018. Available from: https://indiankanoon.org/doc/168671544/.

[4] Supriyo v. Union of India. Writ Petition (Civil) No. 1011 of 2022. Supreme Court of India. 2023. Available from: https://indiankanoon.org/doc/192743429/.

[5] Bhattacharya A, Upadhyay G, Subramanian T, et al. Understanding discrimination against LGBTQIA+ patients in Indian hospitals using a human rights perspective: an exploratory qualitative study. Sex Reprod Health Matters. 2022;29(2):2104678. 10.1080/26410397.2022.2104678. PMC9423841 36017877

[6] Institute of Medicine (US). Committee on Lesbian, Gay, Bisexual, and Transgender Health Issues and Research Gaps and Opportunities. The Health of Lesbian, Gay, Bisexual, and Transgender People: Building a Foundation for Better Understanding. Washington (DC): National Academies Press (US); 2011. 22013611

[7] Obedin-Maliver J, Goldsmith ES, Stewart L, White W, Tran E, Brenman S, et al. Lesbian, gay, bisexual, and transgender-related content in undergraduate medical education. JAMA. 2011;306(9):971–7. 10.1001/jama.2011.1255. 21900137

[8] Arthur S, Jamieson A, Cross H, Nambiar K, Llewellyn CD. Medical students’ awareness of health issues, attitudes, and confidence about caring for lesbian, gay, bisexual and transgender patients: a cross-sectional survey. BMC Med Educ. 2021;21(1):56. 10.1186/s12909-020-02409-6. PMC7809852 33446197

[9] National Medical Commission. Competency Based Undergraduate Curriculum for the Indian Medical Graduate. New Delhi: National Medical Commission; 2018. Available from: https://www.nmc.org.in/information-desk/for-colleges/ug-curriculum/. Cited 2026 May 5.

[10] Agarwal A, Thiyam A. Healthcare, culture & curriculum: addressing the need for LGBT+ inclusive medical education in India. Lancet Reg Health Southeast Asia. 2023;8:100101. 10.1016/S2772-3682(22)00101-9. PMC10305984 37384136

[11] Waghmare P, Mediboina A, Harshita H, Ukey UU, Kherde P. Readiness of medical students and budding doctors about the LGBTQ+ community: a cross-sectional study from India. Cureus. 2024;16(2):e55177. 10.7759/cureus.55177. PMC10980587 38558703

[12] Divya R, Menon A. Transgender healthcare and Indian medical students: is curricular revamping the need of the hour? Chettinad Health City Med J. 2022;11(1):16–20.

[13] Sharma M, Mohlia A, Kukreti P, Saurabh, Kataria D. Sexual and gender minority health care issues related competence and preparedness among health care professionals and trainees in India: a comparative study. SAGE Open Med. 2024;12:20503121241299033. 10.1177/20503121241299033.

[14] Parameshwaran V, Cockbain BC, Hillyard M, Price JR. Is the lack of specific lesbian, gay, bisexual, transgender and queer/questioning (LGBTQ) health care education in medical school a cause for concern? Evidence from a survey of knowledge and practice among UK medical students. J Homosex. 2017;64(3):367–81. 10.1080/00918369.2016.1190218. 27184023

[15] Von Elm E, Altman DG, Egger M, Pocock SJ, Gøtzsche PC, Vandenbroucke JP. The Strengthening the Reporting of Observational Studies in Epidemiology (STROBE) statement: guidelines for reporting observational studies. J Clin Epidemiol. 2008;61(4):344–9. 10.1016/j.jclinepi.2007.11.008. 18313558

[16] Bandura A. Self-efficacy: toward a unifying theory of behavioral change. Psychol Rev. 1977;84(2):191–215. 10.1037/0033-295X.84.2.191. 847061

[17] Sullivan GM, Artino AR Jr. Analyzing and interpreting data from Likert-type scales. J Grad Med Educ. 2013;5(4):541–2. 10.4300/JGME-5-4-18. PMC3886444 24454995

[18] Schmidt AF, Finan C. Linear regression and the normality assumption. J Clin Epidemiol. 2018;98:146–51. 10.1016/j.jclinepi.2017.12.006. 29258908

[19] Hafferty FW. Beyond curriculum reform: confronting medicine’s hidden curriculum. Acad Med. 1998;73(4):403–7. 10.1097/00001888-199804000-00013. 9580717

[20] Kruger J, Dunning D. Unskilled and unaware of it: how difficulties in recognizing one’s own incompetence lead to inflated self-assessments. J Pers Soc Psychol. 1999;77(6):1121–34. 10.1037/0022-3514.77.6.1121. 10626367

[21] Kolb DA. Experiential learning: experience as the source of learning and development. 2nd ed. Upper Saddle River (NJ): Pearson Education; 2015.

[22] Lave J, Wenger E. Situated learning: legitimate peripheral participation. Cambridge: Cambridge University Press; 1991.

[23] Macedo A, Aurindo M, Febra C. Effectiveness of undergraduate medical students training on LGBTQIA+ people health: a systematic review and meta-analysis. BMC Med Educ. 2024;24(1):67. 10.1186/s12909-024-05041-w. PMC10790407 38229060

[24] Nowaskie DZ, Patel AU, Fang RC. A multicenter, multidisciplinary evaluation of 1701 healthcare professional students’ LGBT cultural competency. PLoS ONE. 2020;15(9):e0237670. 10.1371/journal.pone.0237670. PMC7425966 32790797

[25] Pandya A, Redcay A. Access to health services: barriers faced by the transgender population in India. J Gay Lesbian Ment Health. 2021;25(2):146–71. 10.1080/19359705.2020.1850592.

[26] Mayer KH, Bradford JB, Makadon HJ, Stall R, Goldhammer H, Landers S. Sexual and gender minority health: what we know and what needs to be done. Am J Public Health. 2008;98(6):989–97. 10.2105/AJPH.2007.127811. PMC2377288 18445789

