# Supplementary material for: LGBTQ+ healthcare preparedness among undergraduate medical students in India: a cross-sectional study of awareness, attitudes, and clinical confidence
Source: BMC Med Educ. 2026 Aug 13;26:1817. doi: 10.1186/s12909-026-10138-5 (PMC13749769; doi:10.1186/s12909-026-10138-5)
Supplement: Supplementary file 2 [file 12909_2026_10138_MOESM2_ESM.docx]

# ADDITIONAL FILE 2: QUESTIONNAIRE

# Demographic Details

1. Sex
2. Year of study

# Prior Training (Likert scale ranging from 1=Strongly disagree to 5=Strongly agree)

1. I have received specific training on LGBTQ+Q health issues

# Attitude (Likert Scale ranging from 1=Strongly disagree to 5=Strongly agree)

1. LGBTQ+ patients deserve the same level of quality care from health services as heterosexual patients

2. LGBTQ+ patients should only seek health care from LGBTQ+ health clinics

3. LGBTQ+ patients should always disclose their sexual orientation to their physicians

4. I would be comfortable if I became known among my professional peers as a doctor who cares for LGBTQ+ patients

5. I would be comfortable telling my friends and family that I cared for LGBTQ+ patients

6. Same-sex sexual attraction is a natural expression of sexuality in humans

7. It is more challenging to gather a history from an LGBTQ+ patient than of a heterosexual patient

8. It is more challenging to conduct a physical examination of an LGBTQ+ patient than of a heterosexual patient

9. It is more challenging to conduct a genitourinary examination of an LGBTQ+ patient than of a heterosexual patient

10. It is more challenging to discuss sexual behaviour with LGBTQ+ patients than with heterosexual patients

# Awareness (Likert Scale ranging from 1=Very unfamiliar to 5=Very familiar)

1. Are you aware of the terminology - asexual?

2. Are you aware of the terminology - bisexual?

3. Are you aware of the terminology - gay?

4. Are you aware of the terminology - genderqueer?

5. Are you aware of the terminology - intersex?

6. Are you aware of the terminology - lesbian?

7. Are you aware of the terminology - pansexual?

8. Are you aware of the terminology - polyamorous?

9. Are you aware of the terminology - queer?

10. Are you aware of the terminology - trans?

11. Are you aware of the terminology - trans woman?

12. Are you aware of the terminology - trans man?

13. Are you aware of the terminology - transgender?

14. Are you aware of the terminology - transitioning?

15. Are you aware of the terminology - bottom surgery?

16. Are you aware of the terminology - dental dams?

17. Are you aware of the terminology - PEP (post exposure prophylaxis)?

18. Are you aware of the terminology - Phalloplasty?

19. Are you aware of the terminology - neovagina?

20. Are you aware of the terminology - T 'testosterone'?

21. Are you aware of the terminology - Top surgery?

22. Are you aware of the terminology - bareback?

23. Are you aware of the terminology - BDSM?

24. Are you aware of the terminology - bottoming?

25. Are you aware of the terminology - douching?

26. Are you aware of the terminology - fisting?

27. Are you aware of the terminology - rimming?

28. Are you aware of the terminology - serosorting?

29. Are you aware of the terminology - saunas?

30. Are you aware of the terminology - slamming?

31. Are you aware of the terminology - topping?

# Confidence (Likert Scale ranging from 1=Not at all Confident to 5=Very confident)

1. How confident do you feel clarifying unfamiliar sexual or gender terms used by patients?

2. How confident do you feel taking a social history from an LGBTQ+ patient?

3. How confident do you feel taking a sexual history from an LGBTQ+ patient?

4. In a situation where domestic violence would be considered a possibility, how confident do you feel discussing this with an LGBTQ+ patient?

5. How confident do you feel deciding which ward (e.g. male/female ward) a transgender patient should be nursed?

6. How confident do you feel knowing where to look in order to find information about LGBTQ+-specific health services in your area?
