# Supplementary material for: LGBTQ+ healthcare preparedness among undergraduate medical students in India: a cross-sectional study of awareness, attitudes, and clinical confidence
Source: BMC Med Educ. 2026 Aug 13;26:1817. doi: 10.1186/s12909-026-10138-5 (PMC13749769; doi:10.1186/s12909-026-10138-5)
Supplement: Supplementary file 3 [file 12909_2026_10138_MOESM3_ESM.docx]

**Additional File 3**

**Contents**

**Table A1:** Internal consistency of study scales (Cronbach's alpha)

**Table A2:** Item-wise distribution of responses across questionnaire domains (n = 229)

**Table A3:** Post hoc pairwise comparisons of awareness scores across academic years (Dunn's test, Holm-adjusted)

**Table A4:** Shapiro–Wilk test for normality of domain scores (n = 229)

**Figure A1:** Q–Q plots: visual assessment of normality

**Table A5:** Combined normality assessment summary

**Table A6:** Multicollinearity assessment using generalised variance inflation factors (GVIF)

**Supplementary Methods Note**

Domain scores (awareness, attitude, and confidence) deviated from normality as assessed by the Shapiro–Wilk test; accordingly, non-parametric methods (Kruskal–Wallis test with Dunn’s post hoc comparisons and Spearman’s rank correlation) were used for group comparisons and association analyses. Multivariable linear regression was nonetheless applied to identify independent predictors of confidence, given its robustness to moderate violations of normality assumptions at this sample size and to facilitate comparability with existing literature. The normality assumption for linear regression was evaluated by visual inspection of Q–Q plots of regression residuals, which demonstrated no substantial deviation affecting model validity (Figure A1; Tables A3–A4).

**Table A1: Internal consistency of study scales (Cronbach’s alpha)**

| **Scale** | **Items (range)** | **Cronbach’s α** | **Interpretation** |
| --- | --- | --- | --- |
| Awareness | B1–B31 | 0.95 | Excellent |
| Attitude | A1–A10 | 0.80 | Good |
| Confidence | C1–C6 | 0.81 | Good |

Internal consistency was assessed using Cronbach’s alpha. Thresholds applied: ≥0.70 = acceptable; ≥0.80 = good; ≥0.90 = excellent. Reverse scoring was applied to negatively worded attitude items before computation.

**Table A2: Item-wise distribution of responses across questionnaire items**

| **Attitude Domain (n = 229)** | | | | | |
| --- | --- | --- | --- | --- | --- |
| **Questionnaire Item** | **Strongly disagree (%)** | **Disagree (%)** | **Neither disagree nor agree (%)** | **Agree (%)** | **Strongly Agree (%)** |
| LGBTQ+ patients deserve the same level of quality care from health services as heterosexual patients | 0.4 | 2.2 | 11.8 | 25.3 | 60.3 |
| LGBTQ+ patients should only seek health care from LGBTQ+ health clinics | 6.1 | 9.2 | 17.9 | 43.2 | 23.6 |
| LGBTQ+ patients should always disclose their sexual orientation to their physicians | 25.3 | 33.6 | 30.6 | 8.7 | 1.7 |
| I would be comfortable if I became known among my professional peers as a doctor who cares for LGBTQ+ patients | 3.9 | 2.6 | 23.1 | 34.9 | 35.4 |
| I would be comfortable telling my friends and family that I cared for LGBTQ+ patients | 3.9 | 4.4 | 19.7 | 34.1 | 38 |
| Same-sex sexual attraction is a natural expression of sexuality in humans | 8.7 | 5.7 | 21 | 34.1 | 30.6 |
| It is more challenging to gather a history from an LGBTQ+ patient than of a heterosexual patient | 6.1 | 27.9 | 38.9 | 18.8 | 8.3 |
| It is more challenging to conduct a physical examination of an LGBTQ+ patient than of a heterosexual patient | 5.7 | 24.9 | 36.7 | 22.3 | 10.5 |
| It is more challenging to conduct a genitourinary examination of an LGBTQ+ patient than of a heterosexual patient | 7.4 | 22.7 | 41.9 | 20.5 | 7.4 |
| It is more challenging to discuss sexual behaviour with LGBTQ+ patients than with heterosexual patients | 9.6 | 25 | 32 | 24.1 | 9.2 |

| **Awareness Domain (n = 229)** | | | | | |
| --- | --- | --- | --- | --- | --- |
| **Terminology** | **Never heard (%)** | **Heard of it (%)** | **Somewhat Familiar (%)** | **Familiar (%)** | **Very Familiar (%)** |
| Asexual | 2.2 | 3.9 | 20.1 | 38 | 35.8 |
| Bisexual | 2.6 | 1.7 | 17 | 38.9 | 39.7 |
| Gay | 2.6 | 1.3 | 16.2 | 40.2 | 39.7 |
| Genderqueer | 6.6 | 15.3 | 26.6 | 26.6 | 24.9 |
| Intersex | 4.4 | 8.3 | 30.6 | 28.4 | 28.4 |
| Lesbian | 2.2 | 3.1 | 15.3 | 37.6 | 41.9 |
| Pansexual | 7.4 | 10 | 24.5 | 30.1 | 27.9 |
| Polyamorous | 9.2 | 18.8 | 29.7 | 21.8 | 20.5 |
| Queer | 7.9 | 12.2 | 21.4 | 32.3 | 26.2 |
| Trans | 2.2 | 1.3 | 17.5 | 48.5 | 30.6 |
| Trans Woman | 2.6 | 1.7 | 14.4 | 64.6 | 16.6 |
| Trans Man | 2.2 | 3.1 | 15.7 | 62 | 17 |
| Transgender | 2.6 | 2.2 | 14.4 | 62.9 | 17.9 |
| Transitioning | 4.4 | 7.4 | 16.2 | 59 | 13.1 |
| Bottom Surgery | 5.7 | 12.2 | 18.3 | 57.6 | 6.1 |
| Dental Dams | 10.5 | 16.6 | 16.2 | 51.1 | 5.7 |
| PEP (Post Exposure Prophylaxis) | 5.2 | 4.8 | 12.2 | 62.4 | 15.3 |
| Phalloplasty | 10 | 14.4 | 16.6 | 52.8 | 6.1 |
| Neovagina | 7 | 14.8 | 17.9 | 52.8 | 7.4 |
| Testosterone | 2.6 | 6.1 | 13.5 | 60.7 | 17 |
| Top Surgery | 7.4 | 8.3 | 14 | 61.1 | 9.2 |
| Bareback | 10 | 14.4 | 16.2 | 53.7 | 5.7 |
| BDSM | 7 | 11.8 | 19.7 | 52 | 9.6 |
| Bottoming | 7.4 | 16.6 | 17 | 52 | 7 |
| Douching | 6.6 | 14.4 | 17 | 53.7 | 8.3 |
| Fisting | 6.6 | 14.8 | 17.9 | 53.3 | 7.4 |
| Rimming | 7.4 | 14 | 19.2 | 52.8 | 6.6 |
| Serosorting | 10.5 | 19.7 | 17.9 | 48.5 | 3.5 |
| Saunas | 7.9 | 17 | 15.3 | 54.6 | 5.2 |
| Slamming | 9.2 | 15.3 | 16.2 | 52.4 | 7 |
| Topping | 9.2 | 14.8 | 18.3 | 52.4 | 5.2 |

| **Confidence Domain (n = 229)** | | | | | |
| --- | --- | --- | --- | --- | --- |
| **Questionnaire Item** | **Not at all Confident (%)** | **Unconfident (%)** | **Neither unconfident nor confident (%)** | **Confident (%)** | **Very confident (%)** |
| How confident do you feel clarifying unfamiliar sexual or gender terms used by patients? | 7.4 | 10.9 | 41.9 | 28.4 | 11.4 |
| How confident do you feel taking a social history from an LGBTQ+ patient? | 3.5 | 11.8 | 36.7 | 34.5 | 13.5 |
| How confident do you feel taking a sexual history from an LGBT patient? | 3.9 | 10.5 | 36.2 | 34.9 | 14.4 |
| In a situation where domestic violence would be considered a possibility, how confident do you feel discussing this with an LGBT patient? | 4.8 | 5.7 | 38 | 35.4 | 16.2 |
| How confident do you feel deciding which ward (e.g. male/female ward) a transgender patient should be nursed? | 10.5 | 21.4 | 43.7 | 16.6 | 7.9 |
| How confident do you feel knowing where to look in order to find information about LGBT-specific health services in your area? | 11.4 | 25.8 | 41.5 | 14.4 | 7 |

**Table A3: Post hoc pairwise comparisons of awareness scores across academic years (Dunn's test, Holm-adjusted)**

| **Comparison** | **Median (IQR) — Group 1** | **Median (IQR) — Group 2** | **Adjusted p** |
| --- | --- | --- | --- |
| Third year vs Second year | 3.84 (3.03–4.06) | 3.23 (2.85–3.85) | 0.003 |
| Final year vs Second year | 4.00 (3.78–4.25) | 3.23 (2.85–3.85) | <0.001 |
| Final year vs Third year | 4.00 (3.78–4.25) | 3.84 (3.03–4.06) | 0.003 |

Pairwise comparisons following a significant Kruskal–Wallis test (χ² = 38.32, p < 0.001). p-values adjusted using the Holm method. Attitude and confidence domains showed no significant overall difference across years and were therefore not subjected to post hoc testing.

**Table A4. Normality assessment of study variables (Shapiro–Wilk test)**

| **Variable** | **W statistic** | **p-value** | **Interpretation** |
| --- | --- | --- | --- |
| Awareness score | 0.957 | <0.001 | Non-normal |
| Attitude score | 0.985 | 0.016 | Slight deviation from normality |
| Confidence score | 0.979 | 0.002 | Non-normal |

Shapiro–Wilk testing indicated statistically significant deviations from normality across all three domain scores. Given the sample size (n = 229), the test has high statistical power and may detect trivial departures from normality. Findings were therefore considered alongside Q–Q plot inspection (Figure A1) before determining the appropriate analytical strategy.

**Figure A1: Visual assessment of normality (Q–Q plots)**

**Table A5. Integrated assessment of normality and justification of analytical approach**

| **Assessment Method** | **Key Findings** | **Interpretation and Analytical Implication** |
| --- | --- | --- |
| Shapiro–Wilk test (domain scores) | Statistically significant deviation from normality observed across all domains | Indicates true statistical departure from normality; however, this test is highly sensitive to sample size and may not quantify the magnitude or practical relevance of deviation |
| Q–Q plots (raw domain scores) | Deviations primarily observed at distribution tails; central portions of distributions closely approximate the reference line | Suggests that non-normality is limited in extent and unlikely to significantly bias parametric estimates |
| Q–Q plot (regression residuals) | Residuals largely demonstrated close adherence to the reference diagonal without systematic skewness or kurtosis | Confirms that the normality assumption relevant to linear regression (i.e., normality of residuals) is adequately satisfied |
| Sample size considerations (n = 229) | Moderate-to-large sample size | Ensures robustness of linear regression estimates via the central limit theorem, even in the presence of non-normal outcome distributions |
| Overall synthesis | Statistical tests detect non-normality in domain scores, but visual diagnostics and residual analysis indicate acceptable model behaviour | Non-parametric methods were appropriately used for primary analyses; multivariable linear regression was retained for adjusted analyses as its assumptions were met at the level of residuals and estimates are robust |

**Table A6: Multicollinearity assessment using generalised variance inflation factors (GVIF)**

| **Predictor** | **GVIF** | **Df** | **GVIF^1/-2Df^** |
| --- | --- | --- | --- |
| Awareness score | 1.30 | 1 | 1.14 |
| Attitude score | 1.12 | 1 | 1.06 |
| Year of study | 1.26 | 2 | 1.06 |
| Gender | 1.12 | 2 | 1.03 |
| Prior training | 1.06 | 1 | 1.03 |

Adjusted GVIF values ≤1.5 were considered indicative of negligible collinearity.

Multicollinearity was assessed using generalised variance inflation factors (GVIF) as implemented in the car package in R. GVIF¹ᐟ⁻²ᴰᶠ values ≤1.5 are generally considered indicative of negligible collinearity. All predictors in the multivariable model demonstrated GVIF¹ᐟ⁻²ᴰᶠ values ranging from 1.03 to 1.14, confirming the absence of meaningful multicollinearity.
